# Ssc-mir-221-3p regulates melanin production in Xiang pigs melanocytes by targeting the *TYRP1* gene

**DOI:** 10.1186/s12864-023-09451-w

**Published:** 2023-07-01

**Authors:** Wei Yuan, Hai Qin, Huan Bi, Depeng Zhao, Yiyu Zhang, Wei Chen

**Affiliations:** 1 Key Laboratory of Animal Genetics, Breeding and Reproduction in the Plateau Mountainous Region, Ministry of Education, Guizhou University, Guiyang, 550025 Guizhou Province China; 2 Key Laboratory of Animal Genetics, Breeding and Reproduction, Guiyang, 550025 Guizhou Province China; 3 College of Animal Science, Guizhou University, Guiyang, 550025 Guizhou Province China

**Keywords:** Ssc-miR-221-3p, TYRP1, Xiang pigs, Melanocyte, Melanin, Gene family

## Abstract

**Background:**

MicroRNAs (miRNAs) are small endogenous non-coding RNAs that regulate gene expression by down-regulating it. Several studies have suggested that miRNAs plays a crucial role in mammalian skin color production. The *TYRP1* gene, a member of the tyrosine family, is an important candidate gene that affects melanogenesis. This study aimed to identify genes and miRNAs that affect melanin production in Xiang pigs by transcriptome sequencing, and to validate their targeted regulatory relationships.

**Results:**

17 miRNAs and 1,230 genes were significantly differentially expressed (*P* < 0.05) in the black and white skin tissues of Jianbai Xiang pigs. miRNA-221-3p was identified as a candidate miRNA for melanin formation and its target gene, *TYRP1*, was selected. The *TYRP1* gene is a member of the *TYR* gene family, which evolved from the *TYR* gene through chromosome segmental duplication. The function of the gene was highly conserved throughout the evolutionary process. overexpression of *TYRP1* gene significantly increased the expression of *TYR*, *TYRP1*, and *DCT* genes *P* < 0.01, which led to an increase in the relative content of melanin. Silencing of *TYRP1* through the use of TYRP1-siRNA significantly reduced the expression of *TYR*, *TYRP1*, and *DCT* genes in Jianbai Xiang pig melanocytes *P* < 0.01, which in turn decreased the relative melanin content. The targeted binding relationship between ssc-miR-221-3p and *TYRP1* gene was validated. After transfection of porcine melanocytes with ssc-miR-221-3p mimic, the expression of ssc-miR-221-3p was significantly up-regulated (*P* < 0.01). Furthermore, the mRNA and protein levels of *TYR*, *TYRP1*, and *DCT* genes were significantly down-regulated (*P* < 0.01), and melanin content in cells was significantly reduced (*P* < 0.01).

**Conclusion:**

The *TYRP1* gene affects melanogenesis in melanocytes of Jianbai Xiang pigs, and ssc-miR-221-3p targets the *TYRP1* gene to regulate melanogenesis in melanocytes of Jianbai Xiang pigs.

**Supplementary Information:**

The online version contains supplementary material available at 10.1186/s12864-023-09451-w.

## Introduction

The Xiang Pig is a breed, raised in the mountainous regions of China under ecological and cultural protection. The Jianbai Xiang pig belongs to the Xiang pig breed. Due to the unique karst landscape, the Jianbai Xiang Pig has a mainly inbred population, which aids in preserving its distinct genetic makeup. The Jianbai Xiang pig is characterized by black heads and tails with a white middle. Its skin color is a crucial trait that can provide significant benefits in pig production, breeding, and evaluating product quality. Moreover, skin color can also be a reference for determining breed purity and genetic relatedness.

Previous research has demonstrated that the deposition of different types and amounts of melanin in the skin matrix is a primary factor in mammalian skin color phenotype formation [1, 2]. Melanin synthesis and distribution in animals play a crucial role in skin color formation, with two primary types of melanin, eumelanin, which appears as a primarily brown-black or dark color, and pheomelanin, which appears as a reddish-yellow color. Melanocytes, which synthesize melanin, are located mainly in the basal layer of the epidermis. The formation of melanin is a complex process that involves the differentiation, proliferation, migration, and maturation of melanocytes, as well as the synthesis of melanin, which is accompanied by the participation of numerous functional genes. Through the interaction of these genes, a complex regulatory network is formed for the synthesis of melanin [3]. Several mammalian skin color master genes, such as *MC1R* [4], *TYR* family [5] (*TYR*, *TYRP1*, *DCT*), *KIT* [6], *MLANA* [7], *MITF* [8], and *EDNRB* [9], regulate melanin deposition and determine animal skin color.

TYRP1, a tyrosine-related proteinase 1, plays a vital role in melanin formation [10]. It encodes dihydroxyindole carboxylic acid oxidase, while tyrosinase-related protein 2 (TYRP2) encodes dopachrome intercalase (also known as dopachrome isomerase). TYR, TYRP1, and TYRP2 together form a family of tyrosinase-related proteins, where TYR acts as the rate-limiting enzyme for the production of eumelanin and pyomelanin [11], and TYRP1 and TYRP2 are the rate-limiting enzymes for eumelanin production [12].

MicroRNAs (miRNAs) are highly conserved, non-coding single-stranded RNAs of approximately 21–25 nucleotides that act as post-transcriptional regulators. They bind to target mRNAs in a sequence-specific manner, resulting in mRNA degradation or protein translation inhibition, thereby playing a crucial role in complex organismal regulatory processes [13]. Functional miRNA binding sites are commonly found in the target mRNA-3’UTR, but can also be present in the 5’UTR or coding region [14, 15]. When miRNA binding sites in the 3’UTR or CDS region of the target gene have similar sequence and structural properties, they exhibit the same selection intensity and can result in stable interactions between miRNA and the target gene, leading to stronger inhibition of target gene expression [16]. Recent studies have demonstrated that miRNAs regulate critical biological processes, including cancer [17], cardiovascular disease [18], and albinism [19]. Additionally, miRNAs have been found to modulate animal coat color or melanogenesis, as evidenced by the impact of miR-27a on goat coat color, which causes it to change from brown to white by targeting *WNT3A* and *KITLG* [20]. These findings highlight the essential roles played by miRNAs in biological diversity and adaptation.

Transcriptome sequencing technology is a powerful tool for studying the molecular mechanisms underlying melanin synthesis and regulation. By analyzing the expression profiles of coding and non-coding RNAs in different coat color parts of pigs, researchers can identify differentially expressed miRNAs and their target genes involved in melanin biosynthesis, pigmentation, and tyrosine metabolism [21]. This approach can provide insights into the complex regulatory network of melanin synthesis and contribute to the development of therapeutic approaches for pigmentation-related disorders.

This study employed transcriptome analysis to identify candidate genes associated with melanogenesis in Jianbai Xiang Pig skin. The researchers focused on ssc-miR-221-3p and its target gene, *TYRP1*. To better understand *TYRP1*, gene family analysis was conducted to study its evolutionary history. To investigate the molecular mechanism of ssc-miR-221-3p and its role in regulating melanin synthesis in porcine melanocytes, a range of techniques, including cell culture, transfection, dual luciferase reporter assay, qRT-PCR, Western blotting, and melanin content assay were utilized.

The primary objective of this study was to provide insights into the development of functional genes related to melanin deposition in Guizhou Xiang pigs. This could lead to the selection and breeding of Guizhou characteristic pig breeds and offer potential solutions for regulating abnormal melanin production diseases.

## Results and analysis

### Reads mapping to the Xiang pig transcriptome and quality control

To analyze the miRNA and mRNA transcriptome sequencing of black and white skin tissues from Jianbai Xiang pigs. The sequencing data underwent quality control and filtering, with all experimental samples achieving a Q30% of sequencing quality score greater than 94.37%. Clean reads were mapped to the reference genome of pigs. The results indicated an average alignment rate of 67.52% for miRNA and 92.50% for mRNA.

### The candidate ssc-miR-221-3p and *TYRP* 1 gene related to melanin synthesis were screened out based on transcriptome sequencing

**Fig. 1:**
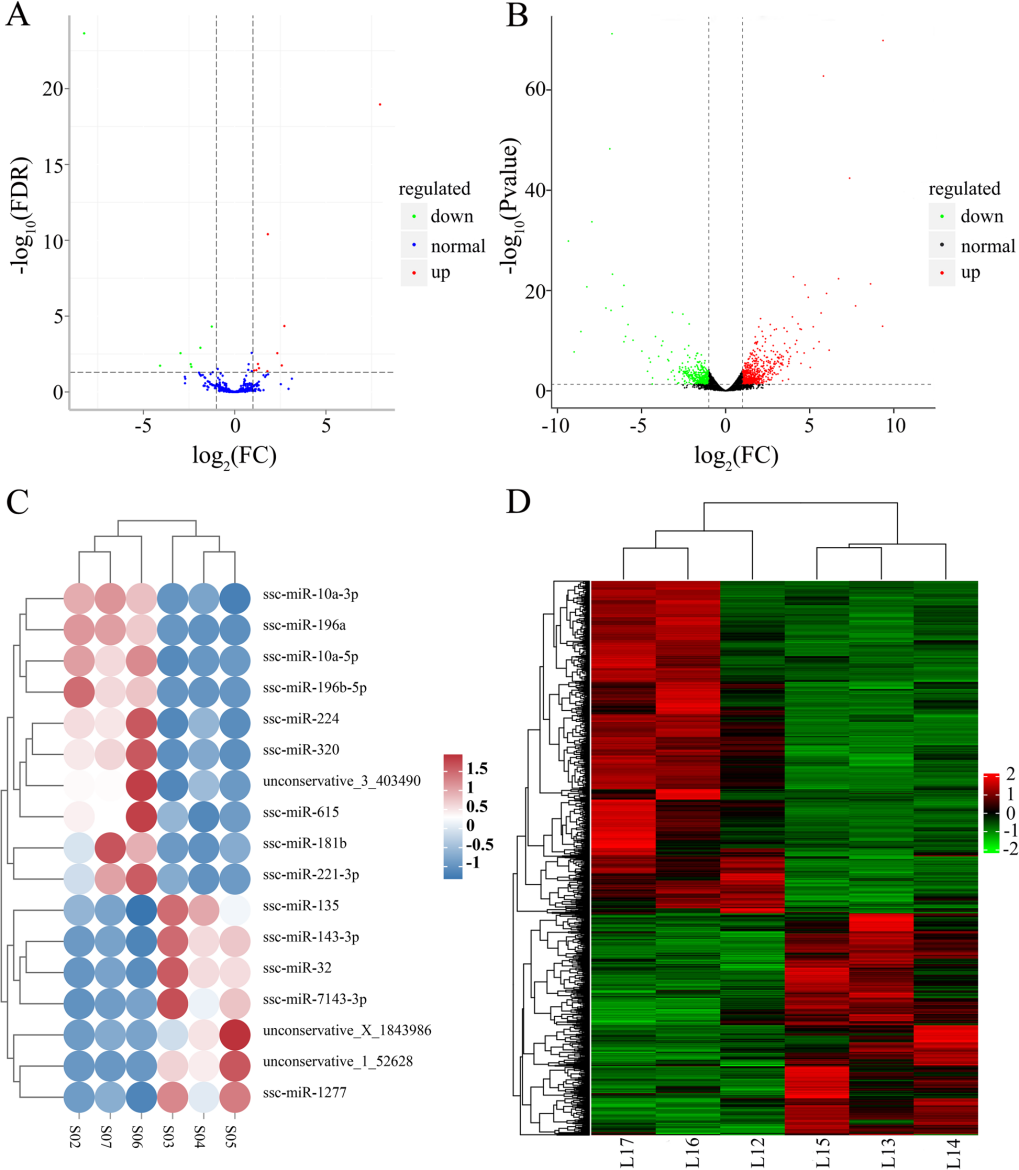
Transcriptome analysis of miRNA and mRNA in the black and white skin tissue of Jianbai Xiang pigs. **A**, **B** The differential expression volcano map for miRNA and mRNA. **C**, **D** miRNA and mRNA differential expression clustering map. Columns represent various samples, rows represent different miRNAs and mRNAs, clustered by log_10_(TPM + 1) and log_10_(FPKM + 0.000001) values, respectively. The color red indicates high expression miRNAs and mRNAs, while green represents low expression miRNAs and mRNAs. The skin samples collected from the black coat on the back of Jianbai Xiang pigs are labeled as S03, S04, S05, L13, L14, and L15. On the other hand, the skin samples from the white coat on the head of Jianbai Xiang pigs are labeled as S02, S06, S07, L12, L16, and L17

A total of 775 miRNAs and 26,229 mRNAs were identified, out of which 17 miRNAs and 1230 mRNAs exhibited significant differential expression between black and white skin tissues in Jianbai Xiang Pig (Supplementary Table S1). 10 miRNAs were significantly up-regulated and 7 miRNAs were significantly down-regulated in white skin tissue compared to black skin tissue. Similarly, 738 mRNAs were significantly up-regulated while 492 mRNAs were significantly down-regulated (Fig. 1). To gain insight into the transcriptome sequencing results related to skin color, GO and KEGG enrichment analyses were conducted. The findings revealed that the melanin synthesis and tyrosine metabolism pathways were enriched with 9 miRNA target genes and 15 differentially expressed genes (Supplementary Table S2).

**Fig. 2:**
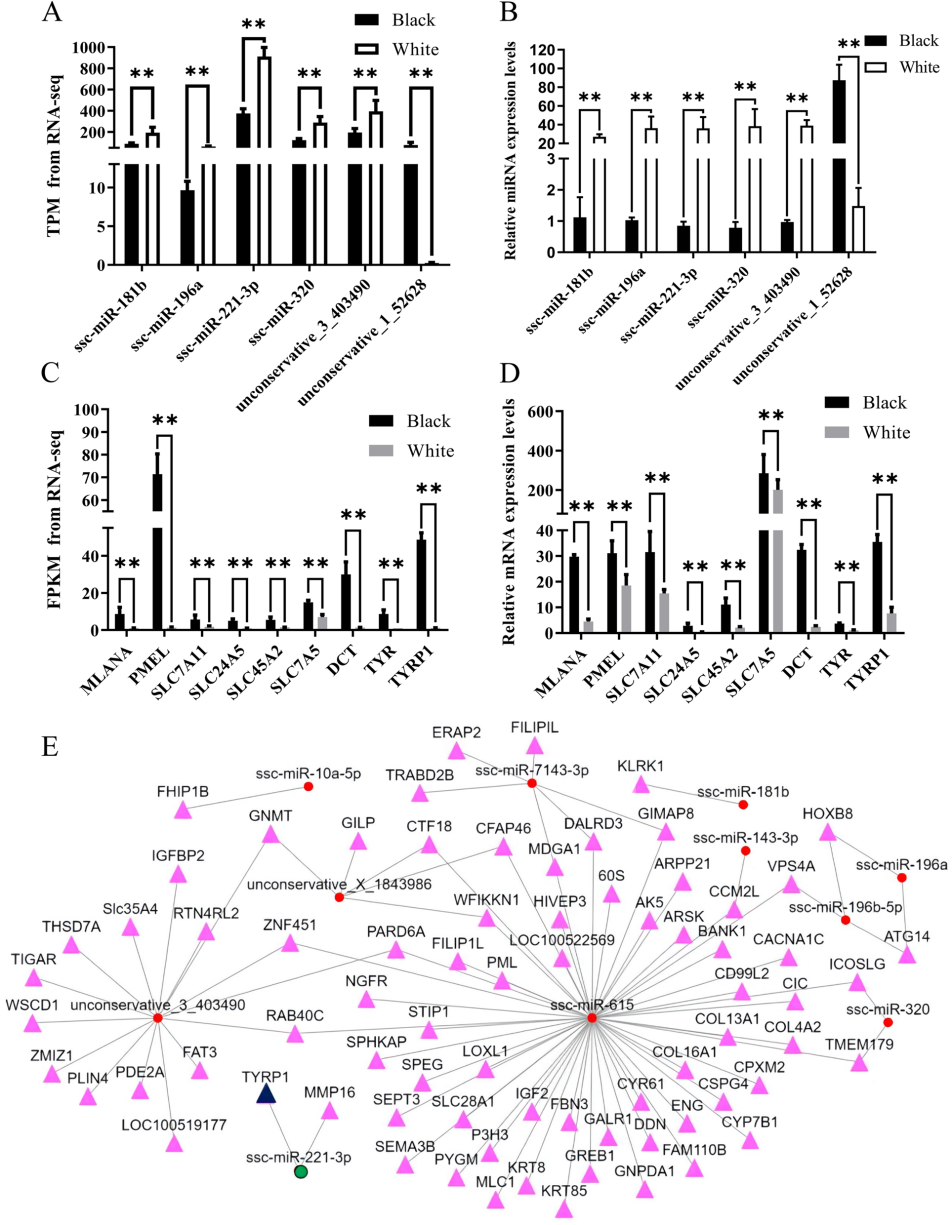
Experiments and correlation analysis. **A**-**D** Expression of selected miRNAs and genes in black and white skin tissue of the Jianbai Xiang pig (**, *P* < 0.001). **A** is the TPM value of each miRNA obtained through transcriptome sequencing, **B** is the miRNA expression of each gene detected by qRT-PCR, **C** is the FPKM value of each gene by transcriptome sequencing, and **D** is the qRT-PCR detection of each gene’s mRNA expression. **E** Differential target genes and miRNAs enriched in melanin synthesis pathway and tyrosine metabolism notification. Triangles and solids represent miRNAs and genes, respectively, while straight lines represented their interactions

To validate the accuracy and reliability of the transcriptome sequencing data, qRT-PCR was performed on a randomly selected of 6 differentially expressed miRNAs (ssc-miR-181b, ssc-miR-196a, ssc-miR-221-3p, ssc-miR-320, unconservative-e_3_403490, unconservative_1_52628) and 9 differentially expressed mRNAs (*SLC24A5*, *SLC45A2*, *SLC7A11*, *SLC7A5*, *MLANA*, *TYR, TYRP1*, *DCT*, *PMEL*). The results showed that the expressing pattern of these 6 miRNAs and 9 genes in black and white skin tissues was consistent with the transcriptome sequencing data (Fig. 2A-D). These findings confirm the accuracy and reliability of the transcriptome sequencing data in this study.

To investigate the correlation between 17 miRNAs exhibiting significant differential expression and 1,230 differentially expressed mRNAs, target genes of differentially expressed miRNAs were intersected with differentially expressed genes. The analysis indicated the presence of 92 target genes of 11 miRNAs in the significantly differentially expressed gene list, as shown in Fig. 2E. White skin tissue demonstrated upregulation of eight miRNAs (ssc-miR-10a-5p, ssc-miR-181b, ssc-miR-196a, ssc-miR-196b-5p, ssc-miR-221-3p, ssc-miR-320, ssc-miR-615, unconservative_3_403490) and downregulation of three miRNAs (ssc-miR-143-3p, ssc-miR-7143-3p, unconservative_X_1843986), compared to black skin tissue. Further analysis of the 92 differentially expressed target genes demonstrated that only the *TYRP1* gene, which is targeted by ssc-miR-221-3p, was enriched in the melanin synthesis pathway. The RNA22 v2 online software predicted that ssc-miR-221-3p could bind to the CDS region of the *TYRP1* gene and regulate its transcription. These findings suggest that miRNA-221-3p may serve as a potential regulatory candidate for melanin synthesis.

### The *TYRP1* gene which regulates skin color production is highly conserved in function during evolution

**Fig. 3:**
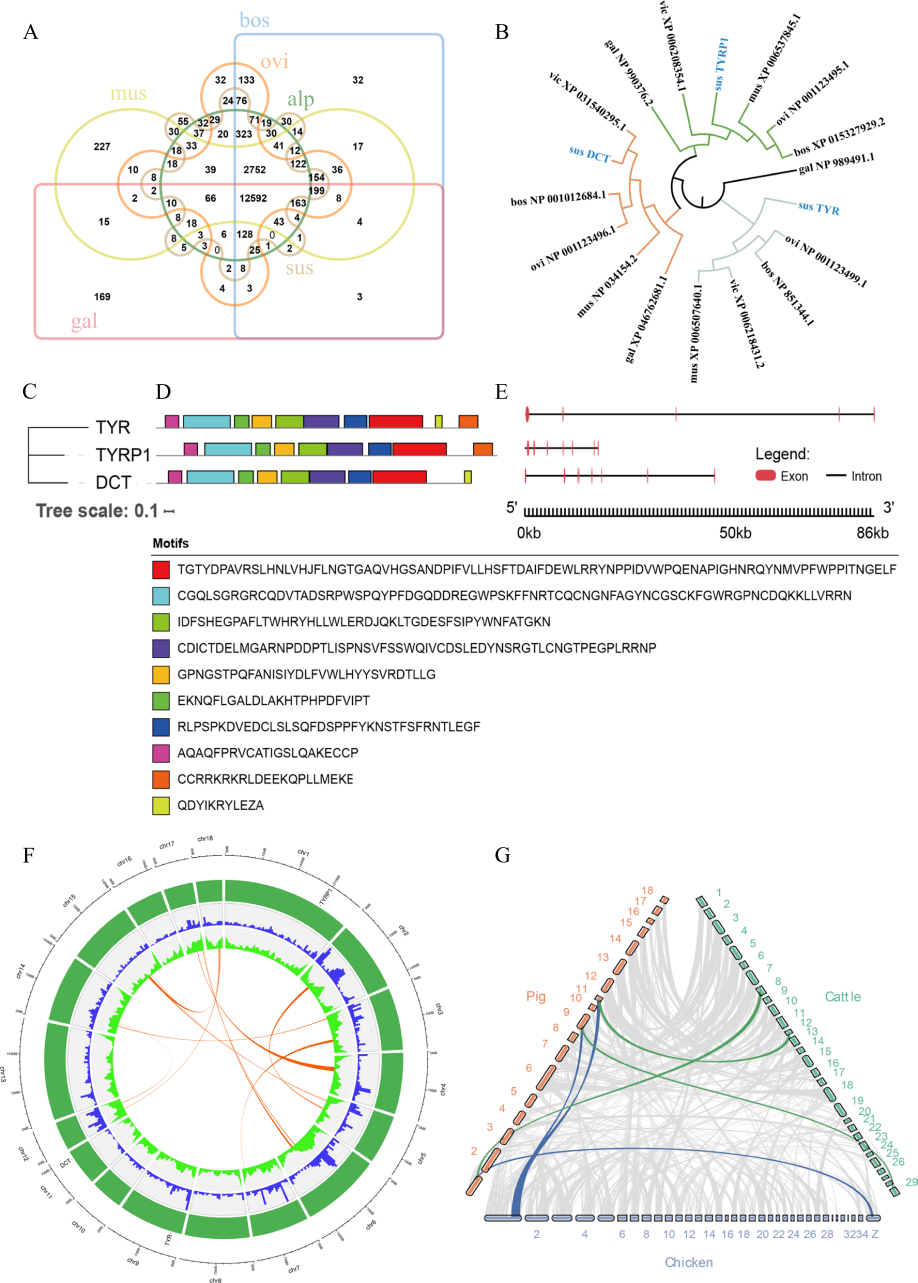
Analysis of the TYR gene family. Refer to the abbreviation directory for the meanings of the abbreviations. **A** Venn diagram of gene families in each species, with the center region indicating gene families that are common to all six species, and the peripheral regions shows gene families that are specific to each species. **B** Highlights members of the *TYR* gene family in pigs with red labels. **C** A phylogenetic tree constructed from the longest protein sequences of the *TYR* gene family members. **D** Ten conserved motifs in the protein sequence. **E** Exons in red regions and introns with lines, and the length of each region is indicated by scale below. **F** The circle plot, with the outermost circos indicating chromosome length and distribution. The second blue bar represents gene density calculated in a 5 Mb sliding window, while the green line in the third circle indicates GC content. The innermost connected circle shows the region of collinearity within the pig species. **G** The collinearity between pig, chicken, and cattle species. The blue line indicates *TYR* gene family pairs between pig and chicken, the green line shows *TYR* gene family pairs between pig and cattle, and the grey section shows collinearity for other genes

This study presents a comprehensive analysis of three *TYR* family genes (*TYR*, *TYRP1*, *DCT*). The TYR family genes, which play a vital role in promoting tyrosine oxidation and melanin production, are highly conserved in pig, cattle, sheep, chicken, alpaca, and mouse (Fig. 3A). The three members of the *TYR* family were confirmed to be present in pig, located on chromosomes 1, 9, and 11, respectively, with no evidence of tandem duplication events (Fig. 3F, Supplementary Fig. S2A). The physicochemical properties of *TYR* family proteins, including TYRP1, TYR, and DCT, were characterized as unstable and hydrophobic (Supplementary Table S3). The *TYRP1* gene, derived from the TYR gene by chromosome structural rearrangement or segmental duplication, has maintained conserved functional domains during evolution(Fig. 3B-E). Collinearity analysis of the *TYR* family genes in pig, cattle, and chicken indicated that the *TYRP1* gene existed during the differentiation of mammals and birds (Fig. 3F, G).

### The *TYRP1* gene affects melanin production in Xiang pig melanocytes

**Fig. 4:**
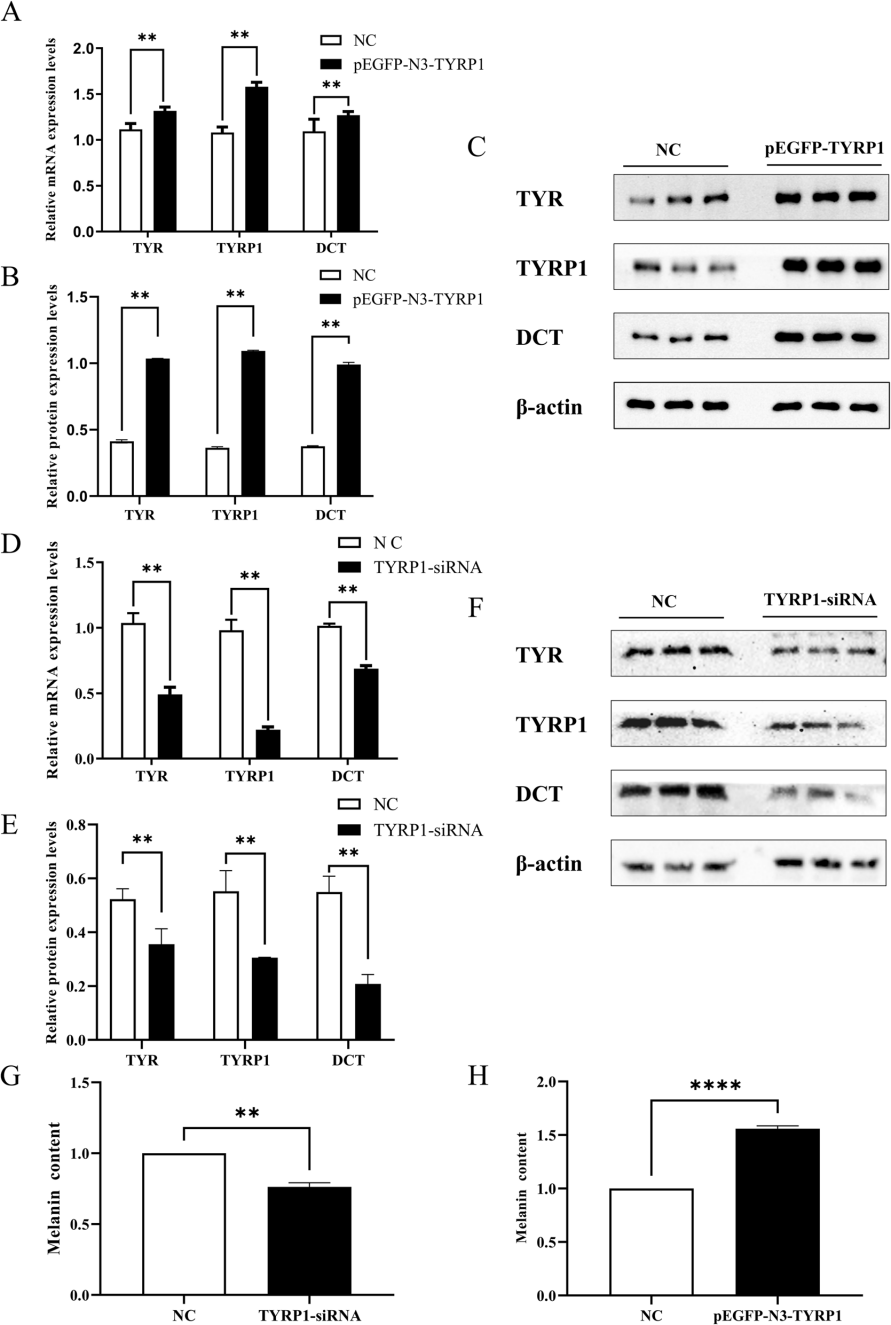
Role of TYRP1 on melanogenesis in melanocytes of Jianbai Xiang pigs (**, *P* < 0.001). **A**-**C** Expression of *TYR*, *TYRP*1, and *DCT* genes in melanocytes after transfection with pEGFP-N3-TYRP1. **D**, **E** Expression of *TYR*, *TYRP1*, and *DCT* genes in melanocytes after transfection with TYRP1-siRNA. **G** Changes in melanin content in melanocytes after transfection with pEGFP-N3-TYRP1. **H** Changes in melanin content in melanocytes after transfection with TYRP1-siRNA (****, *P* < 0.00001)

To investigate the impact of *TYRP1* gene on melanin production in Jianbai Xiang pigs, a pEGFP-N3-*TYRP1* eukaryotic expression vector was constructed and transfected (Supplementary Fig. S3A). The expression of *TYR*, *TYRP1*, and *DCT* genes at both mRNA and protein levels was examined. The findings revealed that overexpression of the *TYRP1* gene resulted in increase the expression of the *TYR* and *DCT* (Fig. 4A-C) and the original electrophoretic gel results were shown in Supplementary Fig. S4.

To determine the interference efficiency of the *TYRP1* gene, five siRNAs of *TYRP1* gene and control siRNA-NC were separately transfected with Jianbai Xiang pig’s melanocytes separately for interference efficiency assay. The results showed that the mRNA expression of the *TYRP1* gene was significantly decreased compared with the control (*P* < 0.01), with the highest interference efficiency of si4-TYRP1 (*P* < 0.01) (Supplementary Fig. S3B). Additionally, 5 siRNAs of the *TYRP1* gene and control siRNA-NC were separately transfected into Xiang pig’s melanocytes, which indicated that reducing the expression of the *TYRP1* gene led to reducing the expression of the *TYR* gene and *DCT* gene (Fig. 4D-F) and the original electrophoretic gel results were shown in Supplementary Fig. S5.

Furthermore, the melanin content was detected using the alkaline solubilization method. It was observed that the melanin content of the pEGFP-N3-TYRP1 transfected group was higher than that of the pEGFP-N3 transfected group, while the melanin content of the TYRP1-siRNA transfected group was lower than that of the Negative Control transfected group.(Fig. 4G, H). These results suggest that the *TYRP1* gene has an impact on melanin production in melanocytes of Jianbai Xiang pigs.

### ssc-miR-221-3p targets the *TYRP1* gene

**Fig. 5:**
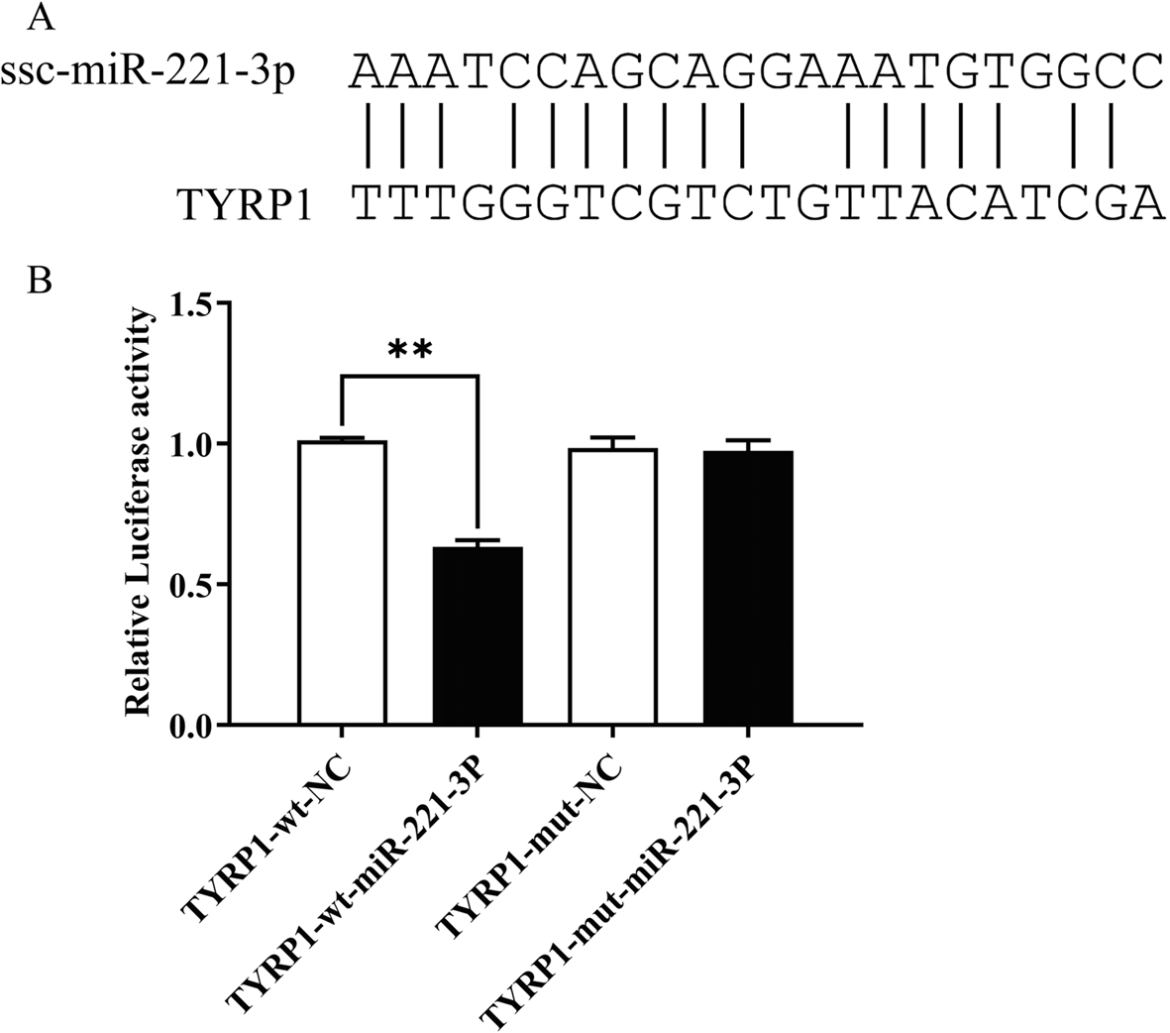
The evidence that ssc-miR-221-3p targets the *TYRP1* gene. **A** The binding site of ssc-miR-221-3p and TYRP1. **B** TYRP1-wt, NC co-transfection group and TYRP1-wt, ssc-miR-221-3p mimic co-transfection group, TYRP1-mut, NC co-transfection group and TYRP1-mut, ssc-miR-221-3p mimic co-transfection group (**, *P* < 0.001)

The online software RNA22v2 predicted that ssc-miR-221-3p may have a binding site to *TYRP1*, suggesting that the *TYRP1* gene may be one of the target genes of ssc-miR-221-3p (Fig. 5A). To confirm this, *TYRP1* dual luciferase reporter vector (pmirGLO-TYRP1 CDS-wt) and mutant vector (pmirGLO-TYRP1 CDS-mut) were cotransfected into 293T cells with ssc-miR-221-3p mimic and Negative Control, respectively, and then subjected to dual luciferase reporter assay. The results indicated that the luciferase activity of the pmirGLO-TYRP1 CDS-wt and ssc-miR-221-3p mimic cotransfected group was significantly reduced by over 30% compared with the pmirGLO-TYRP1 CDS-wt and Negative Control cotransfected group (*P* < 0.01). However, the difference between pmirGLO-TYRP1 CDS-mut and Negative Control cotransfected group did not significantly from the fluorescence activity of pmirGLO-TYRP1 CDS-mt and ssc-miR-221-3p mimic cotransfected group (Fig. 5B). This indicates that ssc-miR-221-3p can target the *TYRP1* gene.

### TYRP1ssc-miR-221-3p targeted to inhibit the expression of TYRP1 gene and then affected melanogenesis in pig melanocytes

**Fig. 6:**
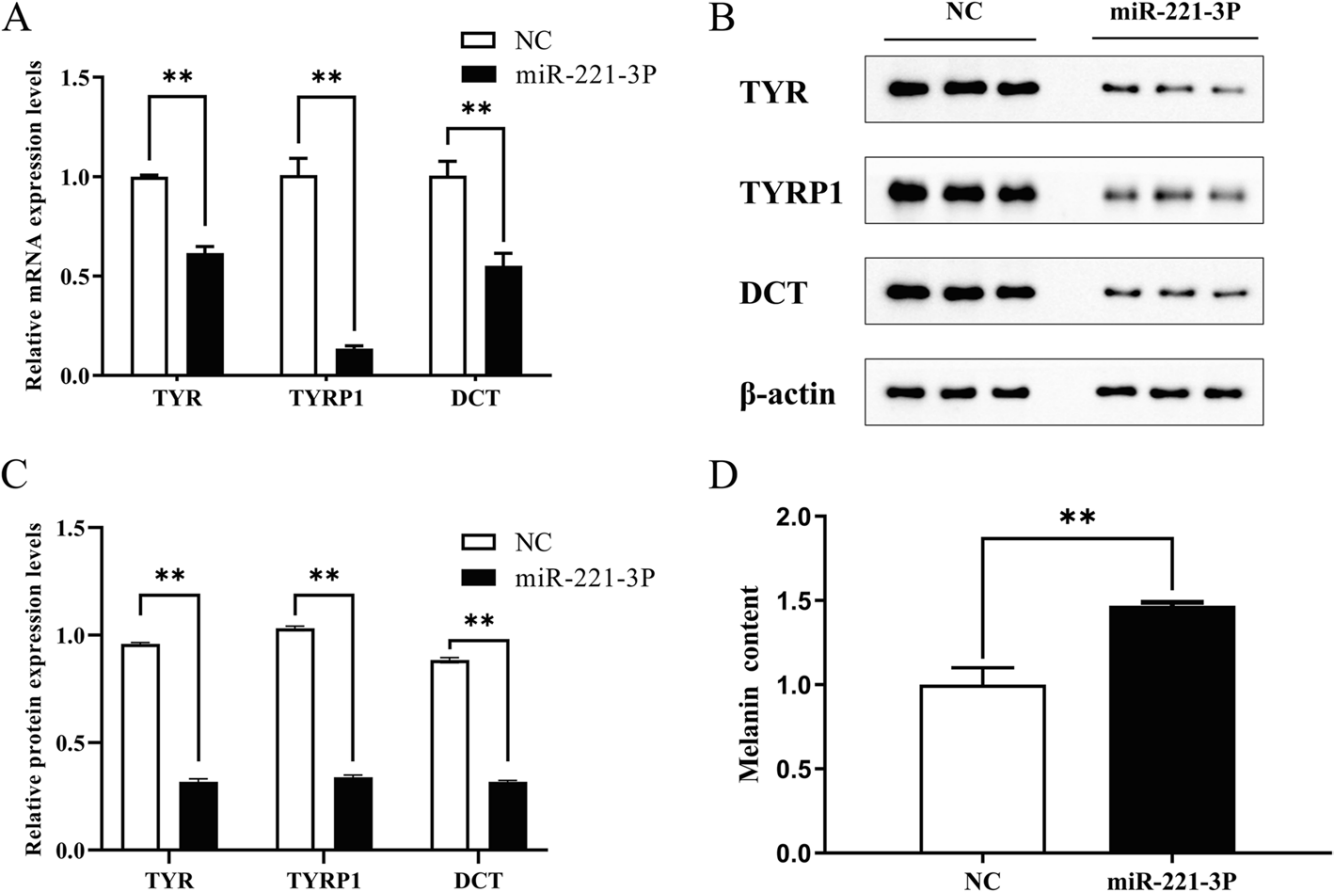
The rusults of an experiment analyzing the impact of ssc-miR-221-3p transfection on the genes related to melanin production. **A** mRNA expression of *TYRP1*, *TYR* and *DCT* following the transfection. **B** Western blotting results of genes involved in melanin production. **C** Protein expression of TYRP1, TYR and DCT in melanocytes post-transfection with ssc-miR-221-3p. **D** Effect of ssc-miR-221-3p transfection on melanin content in melanocytes(**, *P* < 0.001)

After transfection of ssc-miR-221-3p mimic, the expression levels of *TYRP1*, *TYR*, and *DCT* genes were measured using qRT-PCR. Compared with the NC group, the expression levels of *TYRP1*, *TYR*, and *DCT* mRNA were significantly reduced (Fig. 6A). It was discovered that ssc-miR-221-3p mimic could inhibit *TYRP1* gene expression. Furthermore, the effect of ssc-miR-221-3p overexpression on melanogenesis-related genes was examined by Western blotting (Fig. 6B) and the original electrophoretic gel results were shown in Supplementary Fig. S6. The results showed that expression levels of TYRP1, TYR, and TYRP2 protein were significantly reduced in the ssc-miR-221-3p group compared to the NC group (Fig. 6C), These findings suggest that ssc-miR-221-3p mimic could inhibit the expression of TYRP1 protein.

To investigate the regulatory role of ssc-miR-221-3p mimic on melanin production, melanocytes from ssc-miR-221-3p mimic group and NC group were collected and counted separately, and the relative content of melanin was calculated. The results showed that the relative content of melanin in the ssc-miR-221-3p group decreased by 67.3% compared to that in the NC group, indicating that ssc-miR-221-3p mimic inhibited the expression of the *TYRP1* gene and regulated melanin production in melanocytes of Xiang pigs by targeting action (Fig. 6D).

## Discussion

The determination of mammalian skin color involves multiple genes that regulate melanin production, melanocyte production, proliferation, and migration. Melanin production is influenced not only by various genes and signaling pathways but also by an intricate network of miRNAs that interact to form a complex regulatory system [3]. Biotechnology advances have enabled the use of RNA sequencing to study mammalian coat color transcription. For instance, a comparative transcriptome analysis of black and white mink skin identified candidate genes, such as *TYRP1* and *PMEL*, that affect coat color formation [22]. In addition, whole genome sequencing of 542 sheep revealed the identification of the coat color candidate gene, *TYRP1*, in proximity to important SNPs [23]. In this study, transcriptome sequencing of black and white skin tissues of Xiang pigs was employed to screen for the candidate miRNA-221-3p for melanogenesis and its target gene, *TYRP1*.

The *TYR* gene family comprises three members, *TYR*, *TYRP1*, and *TYRP2*, which encode key enzymes involved in melanin synthesis and are present in both prokaryotes and eukaryotes [24]. These genes are important for determining coat color in several species, including pigs, cattle, sheep, chicken, alpaca, and mouse. In Xiang pigs, three members of the *TYR* gene family (*TYR*, *TYRP1*, and *DCT*) were identified using the Hidden Markov Model. Evolutionary studies have suggested that tyrosinase gene arose before the cephalopod-vertebrate divergence and underwent duplication to produce the *TYRP1* and *TYRP2* genes [25]. The results of the present study, which included the construction of intraspecific phylogenetic trees using protein sequence files, indicate that *TYRP1* may have evolved from the same ancestor in different animals and that chromosomal structural rearrangements or segmental duplication have contributed to the evolution of the TYR gene family’s paralogous homologs, *TYRP1* and *DCT*.

Zhao et al. [26] utilized a retroviral vector to insert *TYRP1* cDNA and transfected TYRP1 protein into transcription-deficient melanoma cells. They observed a significant increase in tyrosine hydroxylase and dopa oxidase activities, indicating the effect of TYRP1 on TYR activity. Similarly, Cargill et al. [27] suggested that the *TYRP1* gene may be linked to black and brown spots on a white background in Dalmatian dogs. Ko et al. [28] also found that the *TYR* gene and *TYRP1* gene were expressed at higher levels in black-feathered ducks than in white-feathered ducks. Recent studies on the human *TYRP1* gene have demonstrated that it has *DHICA* oxidase activity and plays a crucial role in the downstream pathway of melanin synthesis [29]. In the current study, overexpression of the *TYRP1* gene in melanocytes of Xiang pigs led to an increase in the relative amount of melanin in melanocytes, while silencing the *TYRP1* gene resulted in a significant decrease the relative amount of melanin in cells. These findings further support the notion that *TYRP1* regulates melanin production in melanocytes.

Previous research has shown that miR-211-3p can exhibit antiviral effects by targeting viral RNAs and promote apoptosis by suppressing *HMBOX1* expression in pigs infected with avian influenza virus [30]. Additionally, in porcine satellite cell expression, miR-221-3p is associated with cell proliferation as an upregulated central miRNA and its central gene, *RUNX1T1* [31]. However, limited studies have examined the role of miR-211-3p in human or other animal skin color. In this study, it has been verified that ssc-miR-221-3p targets the *TYRP1* gene, which is responsible for encoding a crucial enzyme in melanin synthesis. Overexpression of miR-221-3p leads to a reduction in both transcriptional and translational levels of *TYRP1* gene, resulting in a decrease in melanin within cells. Therefore, miR-212-3p can play an significant role in regulating the expression of *TYRP1* and the melanogenesis of melanocytes in Xiang pigs.

## Conclusion

This study comprehensively analyzed the roles of *TYRP1* gene and ssc-miR-221-3p in regulating melanin production in Jianbai Xiang pigs melanocytes. The results indicated that *TYRP1* gene, as a member of the *TYR* gene family, originated from *TYR* gene through chromosomal rearrangement or segmental duplication, and has existed since the differentiation of mammals and birds. The expression of *TYRP1* gene can regulate melanin production and control skin color development. Moreover, ssc-miR-221-3p was identified as a novel regulatory factor of *TYRP1*, which targets and inhibits the expression of *TYRP1* gene, leading to a decrease in melanin content in Jianbai Xiang pig’s melanocytes.

## Materials and methods

### Tissue samples

Skin tissue samples were collected from three two-year-old female Xiang pigs raised on the same farm located in the northeast region of Guizhou province. Xiang pigs were raised under identical feeding conditions to ensure the fulfillment of their nutritional requirements. The white epidermis on the back and black epidermis on the ear of the Jianbai Xiang pig were collected with a skin sampler. The samples were immediately placed in a frozene tube and liquid nitrogen tank. All the epidermis samples were returned to the laboratory and stored in a -80℃ refrigerator. 1.5-month-old Jianbai Xiang pigs were used for cell culture and melanin extraction.

### RNA extraction, library preparation and sequencing

Total RNA was extracted from porcine skin tissue using TRIZOL RNA extraction kits. The purity of the RNA samples was checked using a Nano Drop 2000 spectrophotometer (Thermo Fisher Scientific, Wilmington, DE), ensuring an OD260/280 ≥ 1.8 and OD260/230 ≥ 1.0, indicating high purity RNA suitable for downstream analysis. The concentration of total RNA was determined using a Qubit 2.0 fluorometer and the RNA integrity was assessed using an Agilent 2100 Bioanalyzer (Agilent, Santa Clara-CA, USA), with all samples having RNA Integrity Number (RIN) ≥ 8 and 28 S/18S ≥ 1.5, indicating high-quality RNA. Any potential genomic DNA contamination was eliminated using RNase-free DNase. The qualified total RNA was used to construct both long-chain RNA and small RNA libraries.

After passing the library quality control, mRNA was sequenced on the Illumina HiSeq platform with paired-end (PE) 150 bp reads, while miRNA was sequenced on the Illumina X-ten platform with single-end (SE) 50nt reads. Details of the Xiang pig mRNA and miRNA sequencing data are shown in Supplementary Table S8. *Sus Scrofa 11.1* was used as a reference for sequence alignment and subsequent analysis, which can be obtained from Ensembl (http://www.ensembl.org/Sus_scrofa/Info/Index). For mRNA sequencing, HISAT2 (v.2.2.1) was used to align reads to the reference genome, and StringTie (v.2.1.1) was used to assemble the aligned reads. For miRNA sequencing, Bowtie (v.2.4.1) was used to align the reads to the reference genome. Alignment information to the reference genome is presented in Supplementary Table S9.

### Screening of melanogenesis-related miRNAs and mRNAs in Xiang pigs

Skin tissue samples from black and white coated Xiang pigs were analyzed using the miRBase database (v21) [32] gene annotation information to identify known miRNAs and predict new miRNAs with the mirdep2 software [33]. miRNA expression profiles were constructed using this data. Additionally, mRNA expression profiles were constructed using edgeR software [34] for black and white coated skin tissues separately. Use DEGseq software [35] to detect differentially expressed miRNA and select |log_2_(FC)| ≥ 1 and FDR ≤ 0.05 as the filtering criteria. Use edgeR software to detect differentially expressed mRNA and select |log_2_(FC)| ≥ 1 and *P* value < 0.05 as the filtering criteria.

Target gene prediction of differentially expressed miRNAs was performed using miRanda [36] and RNAhybrid [37]. The intersection of both prediction results was selected, and the predicted target genes were compared to GO and KEGG databases using R package clusterProfiler for enrichment analysis [38]. Target genes of differentially expressed miRNAs were intersected with differential mRNAs from mRNA transcriptome analysis to identify miRNAs and mRNAs that highly correlated with melanogenesis in Jianbai Xiang pigs. miRNA-mRNA regulatory networks were visualized using Omicshare tools online software(https://www.omicshare.com/tools).

### Cell culture and transfection

The laboratory successfully isolated, identified, cultured, and preserved third-generation Jianbai Xiang pig melanocytes and 293T cells. 293T cells were cultured with Dulbecco’s Modified Eagle Medium (Gibco, Beijing, China), and melanocytes were cultured in Melanocyte Medium (ScienCell Research Laboratories, USA), both supplemented with 10% fetal bovine (FBS, Gibco, USA) and 1% penicillin-streptomycin (P/S, Solarbio Life Sciences, Beijing, China). The cells were incubated at 37 °C and 5% CO_2_ in a humidified incubator.

Five pairs of siRNA sequences (Supplementary Table S4) were synthesized by Shanghai Jima Biotechnology Co., Ltd. based on the full sequence of the pig *TYRP1* gene (GenBank No. 537,161). The siRNA with the highest silencing efficiency was selected and transfected into Jianbai Xiang pig melanocytes using the Lipofectamine™ 2000 transfection kit (Invitrogen, USA). The Dual luciferase reporter vectors (TYRP1-wt-F and TYRP1-wt-R) were constructed by Guizhou Hongdal Biotechnology Co., Ltd. (Supplementary Table S5), and miRNA-221-3p mimic, inhibitor, and negative control (NC) were constructed by Shanghai Gene Pharma Co., Jianbai Xiang pig melanocytes were transfection with ssc-miR-221-3p/NC by reached 60%~75% confluency, and 3 replicates were set up in each group.

### Dual luciferase reporter assay

For all luciferase experiments, the melanocytes and 293T cells were transfected and cultured for 72 h. After washing three times with PBS, the luciferase activity was measured using a dual-luciferase reporter gene assay kit (Jiangsu Keygen Biotechnology Co., Ltd., China). The firefly luciferase values were normalized to the sea kidney luciferase value, and the ratio of firefly/sea kidney values was calculated and presented.

### qRT-PCR method

After 48 h transfection period, total RNA was extracted from cells using the Trizol method, followed by cDNA synthesis using HiFiScript cDNA Synthesis Kit (Purdy Bio, Shanghai, China). SYBR Green kit (Tiangen Bio, Beijing, China) was utilized to detect miRNA expression levels, and 2×Taq MasterMix (Kangwei Century Bio, Beijing, China) was used to detect mRNA expression levels, according to the kit instructions. The qRT-PCR reaction system and reaction conditions varied (Supplementary Table 6). The qRT-PCR technique was employed to determine the changes in mRNA expression levels of skin color-related genes in Jianbai Xiang pig melanocytes transfected with pEGFP-N3-*TYRP1* and TYRP1-siRNA. The ssc-miR-221-3p, *TYR*, *TYRP1*, *DCT*, and 5 S rRNA internal reference primer sequences were shown in Supplementary Table S7.

### Western blotting

The total protein of Jianbai Xiang pig melanocytes was extracted using RIPA lysate buffer, and the protein concentration was determined using the BCA method. Subsequently, 10% SDS-PAGE gel electrophoresis was performed, and the proteins were transferred to a polyvinylidene difluoride (PVDF) membrane obtained from Thermo Fisher Scientific, USA. The PVDF membranes were blocked with 5% nonfat dry milk in TBST (CWBIO, Beijing, China) for 3 h. Primary rabbit polyclonal antibodies, including TYR, TYRP1, and DCT (ABclonal, Wuhan, China) and rabbit anti-β-actin (ABclonal, Wuhan, China), were added at a dilution of 1:2,000 for β-actin, 1:1,500 for TYRP1, 1:1,500 for TYR, and 1:1,500 for DCT, and then incubated overnight at 4 ℃. The membranes were then washed 6 times for 5 min each, followed by the addition of goat anti-rabbit secondary antibody labeled with horseradish peroxidase (HRP) (ABclonal, Wuhan, China) at a dilution of 1:10,000, and incubated at 37℃ for 2 h. After membrane washing, the target bands were visualized using High-sig ECL Western Blotting Substrate (Tanon, Shanghai, China). The expression levels of the target proteins were analyzed by Image J software.

### Determination of total melanin content in melanocytes of Xiang pigs

After 48 h of transfection, the Xiang pig melanocytes were washed with PBS, digested, and counted. The cell mass was then lysed in 300 µL of 1 mol/L NaOH solution, and the melanin was dissolved by heating the samples in a metal bath at 80 ℃ for 30 min. The lysed samples were then added to a 96-well enzyme labeling plate (100 µL/well, repeated 5 times) along with melanocyte samples. The absorbance value was measured at 475 nm using a microplate reader (Bio-Tek Synergy HT4, USA). The change of melanin content was analyzed and calculated.

### Analysis of the *TYR* gene family

The *TYR* gene family was screened and classified using a Hidden Markov model (PF00264) from the public database Pfam (https://www.ebi.ac.uk/interpro/) with the HMMER software (v.3.3.2) [39]. The chromosomal distribution of the gene family was determined using TBtools (v.1.082) [40]. Multiple sequence alignment was performed using MUSCLE (v.5.1) [41], and a systematic evolutionary tree was constructed with IQTREE (v.2.0) [42]. The tree was visualized using the iTOL online tool (https://itol.embl.de/). The conservative domain analysis was conducted using the MEME local software (v.5.2.0) [43], and the gene structure diagram was generated using the GSDS online tool (http://gsds.gao-lab.org/). Collinearity analysis was performed using MCScanX and Circos software. The utilization of Latin binomial nomenclature for species is deemed dispensable in the analysis of gene families. Examples of commonly studied domestic animals include the pig (*Sus scrofa*), cow (*Bos taurus*), sheep (*Ovis aries*), chicken (*Gallus gallus*), alpaca (*vicugna pacos*), and mouse (*Mus musculus*).

### Data processing and analysis

The qRT-PCR data were analyzed using the 2^−ΔΔCt^ method. The target bands obtained by Western Blot were scanned and semi-quantitatively analyzed by determining the grayscale values of the target proteins using Image-ProPlus 6.0 software. Processing numbers were analyzed using Excel tables, and data were expressed as Means ± SD. Statistical analysis was performed using one-way ANOVA test with SPSS 19.0 statistical software. Expression variability between two samples was determined using t-test, where * indicates *P* < 0.05 means significant difference and ** indicates *P* < 0.01 means highly significant difference. The experiments were performed in triplicate (*n* = 3) and results were plotted using Prism software.

## Supplementary Information

**Additional file 1: Table S1.** Significantly differentially expressed miRNAs between black and white coated skin tissues of Jianbai Xiang pigs. **Table S2.** KEGG pathway related list of miRNA target genes and genes with significant differential expression enriched in melanogenes. **Table S3.** Physicochemical properties of TYR gene family members. **Table S4.** Fragment details for siRNA. **Table S5.** miRNA-221-3P and mRNAs primer sequences. **Table S6.** Different qRT-PCR reaction systems and reaction conditions for miRNA and mRNA. **Table S7.** Primer sequences for qRT-PCR. **Table S8.** Details of the xiang pig mRNA and miRAN sequencing data. **Table S9.** Alignment information to the reference genome.

**Additional file 2: Fig. S1.** (A) GO rich cluster diagram of differently expressed genes. (B, C) Analysis of differently expressed miRNAs predicting target genes GO and KEGG. **Fig. S2.** (A) Location of TYRP1, TYR, and DCT on the chromosome. (B) Conserved regions are in blue and non-conserved regions are in red. **Fig. S3.** (A) pEGFP-N3-TYRP1 vector double digestion verification. (B) Detection of silencing efficiency of TYRP1 gene in Xiang pig melanocytes.

## Data Availability

The publication contains all data generated or analyzed during the study, including supplementary information files. Additionally, the raw sequencing data is available in the NCBI database with the miRNA BioProject number PRJNA869578 and the mRNA BioProject number PRJNA903765.

## Abbreviations

sus: pig
mus: mouse
bos: cow
ovi: sheep
gal: chicken
alp or vic: alpaca
