# Supplementary material for: Ssc-mir-221-3p regulates melanin production in Xiang pigs melanocytes by targeting the TYRP1 gene
Source: BMC Genomics. 2023 Jul 1;24:369. doi: 10.1186/s12864-023-09451-w (PMC10314631; doi:10.1186/s12864-023-09451-w)
Supplement: Supplementary file 2 — Additional file 2: Fig. S1. (A) GO rich cluster diagram of differently expressed genes. (B, C) Analysis of differently expressed miRNAs predicting target genes GO and KEGG. Fig. S2. (A) Location of TYRP1, TYR, and DCT on the chromosome. (B) Conserved regions are in blue and non-conserved regions are in red. Fig. S3. (A) pEGFP-N3-TYRP1 vector double digestion verification. (B) Detection of silencing efficiency of TYRP1 gene in Xiang pig melanocytes. [file 12864_2023_9451_MOESM2_ESM.pdf]

Contents

Fig S1 (A) GO rich cluster diagram of differently expressed genes. (B, C) Analysis of differently expressed miRNAs predicting target genes GO and KEGG.

Fig S2 (A) Location of *TYRP1*, *TYR*, and *DCT* on the chromosome. (B) Conserved regions are in blue and non-conserved regions are in red.

Fig S3 (A) pEGFP-N3-TYRP1 vector double digestion verification. (B) Detection of silencing efficiency of *TYRP1* gene in Xiang pig melanocytes.

Fig.S4 The original electrophoretic gel results of TYR, TYRP1 and DCT protein expression in melanocytes after transfection with pEGFP-N3-TYRP1. (A) TYR. (B) TYRP1. (C) DCT. (D) β-actin.

Fig.S5 The original electrophoretic gel results of TYR, TYRP1 and DCT protein expression in melanocytes after transfection with TYRP1-siRNA. (A) TYR. (B) TYRP1. (C) DCT. (D) β-actin.

Fig.S6 The original electrophoretic gel results of TYR, TYRP1, and DCT protein expression in melanocytes after transfection with ssc-miR-221-3P. (A) TYR. (B) TYRP1. (C) DCT. (D) β-actin.

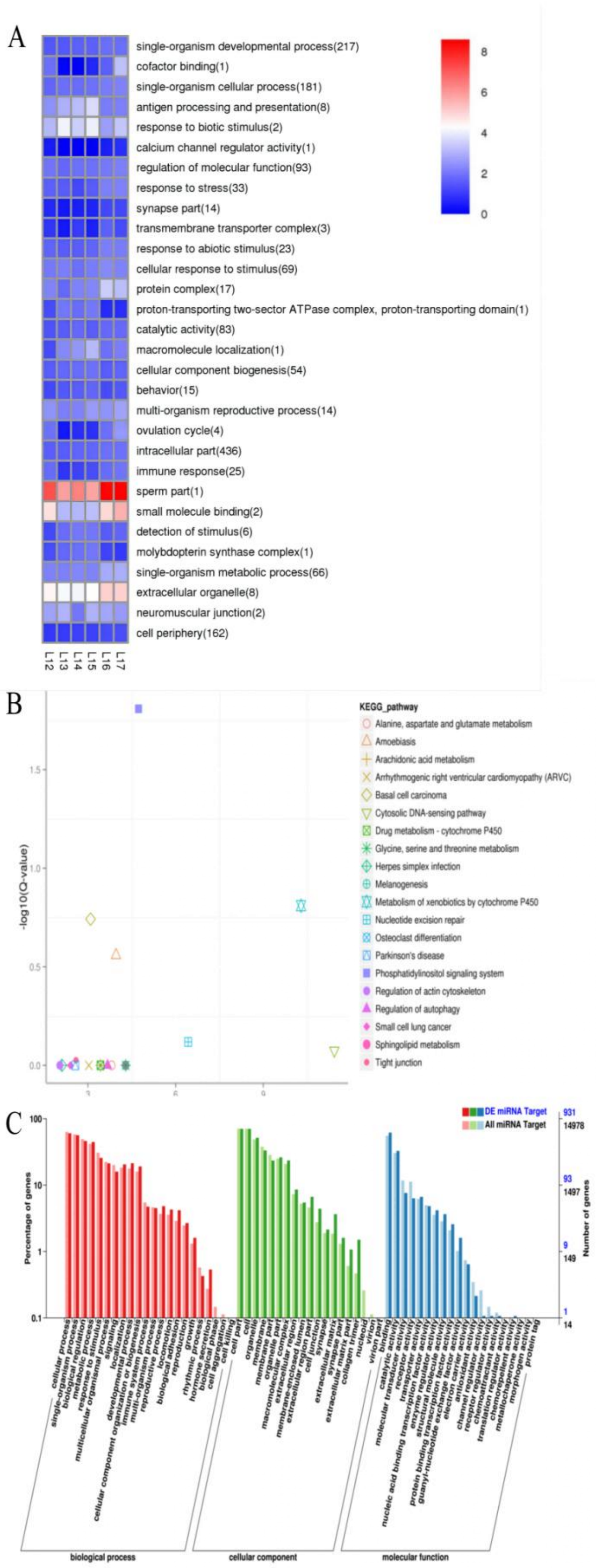

Fig S1 (A) GO rich cluster diagram of differently expressed genes. (B, C) Analysis of differently expressed miRNAs predicting target genes GO and KEGG.

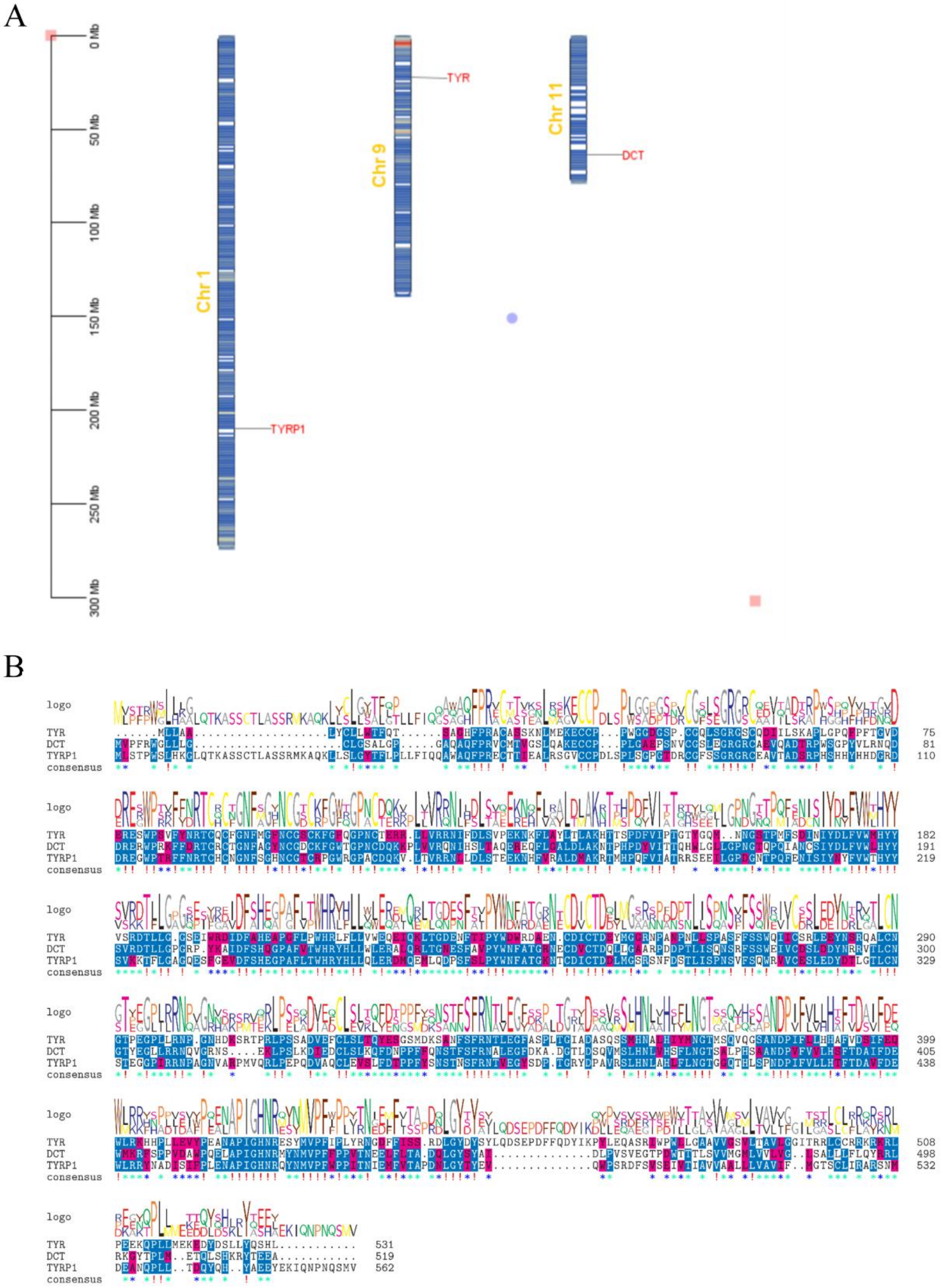

Fig S2 (A) Location of *TYRP1*, *TYR*, and *DCT* on the chromosome. (B) Conserved regions are in blue and non-conserved regions are in red.

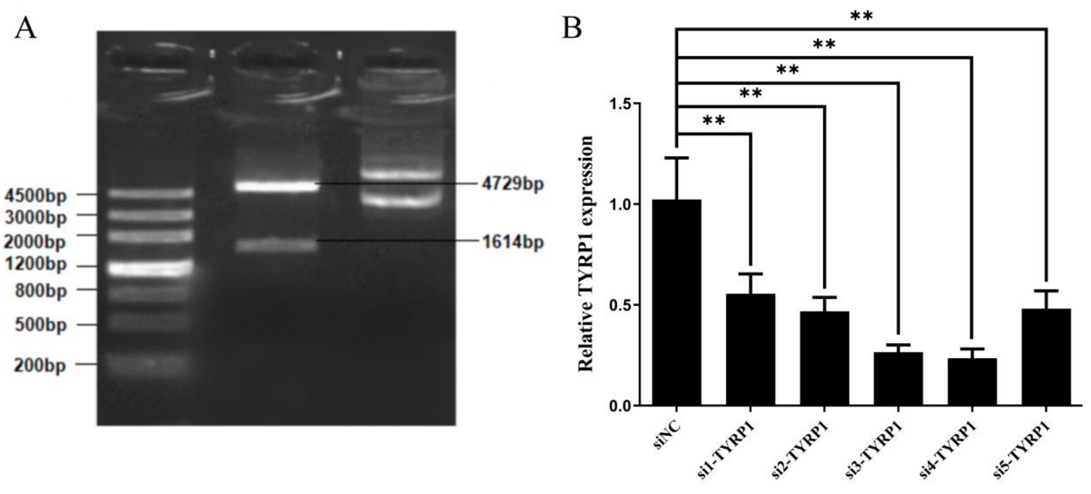

Fig S3 (A) pEGFP-N3-TYRP1 vector double digestion verification. (B) Detection of silencing efficiency of *TYRP1* gene in Xiang pig melanocytes.

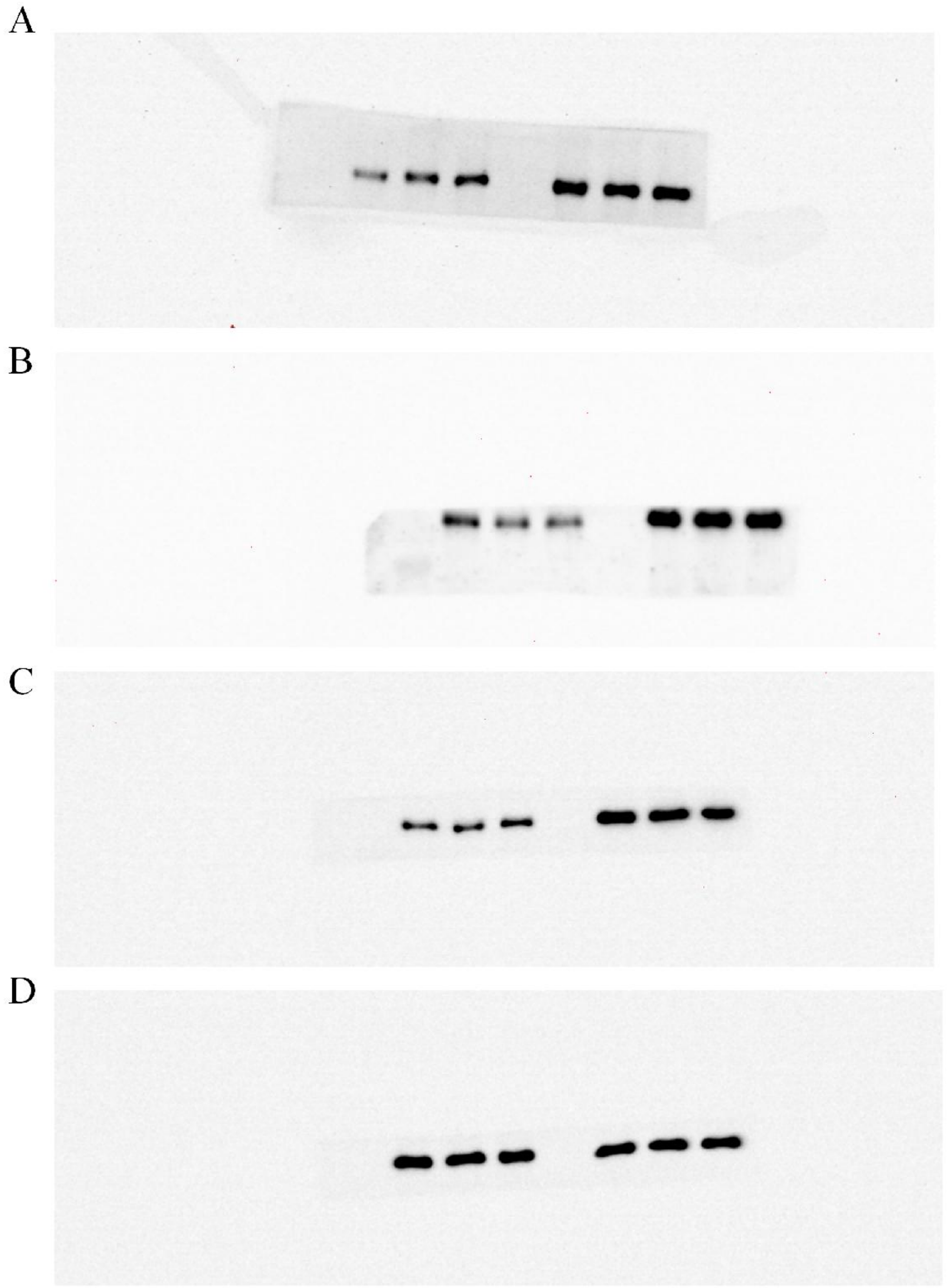

Fig.S4 The original electrophoretic gel results of TYR, TYRP1 and DCT protein expression in melanocytes after transfection with pEGFP-N3-TYRP1. (A) TYR. (B) TYRP1. (C) DCT. (D) β-actin.

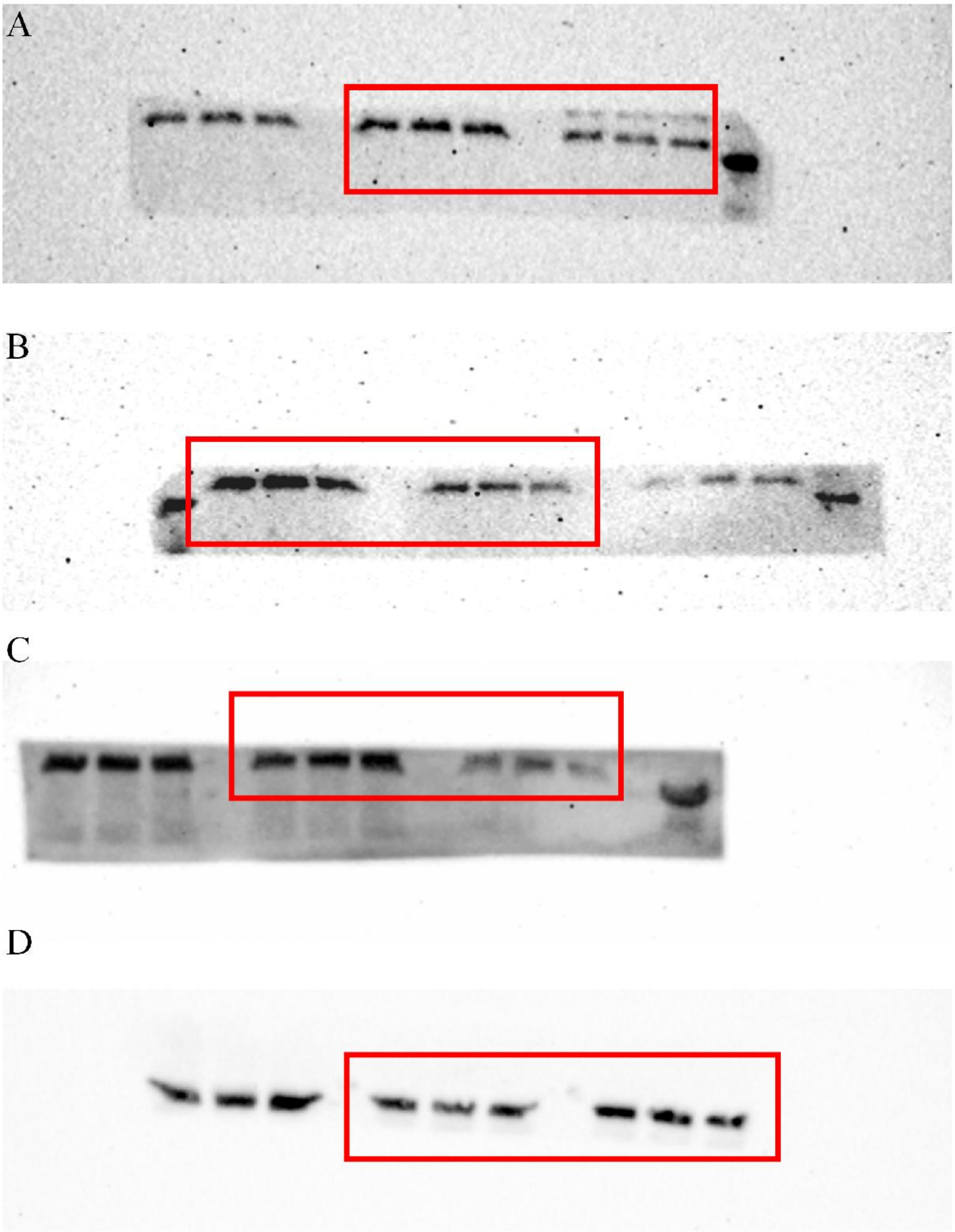

Fig.S5 The original electrophoretic gel results of TYR, TYRP1 and DCT protein expression in melanocytes after transfection with TYRP1-siRNA. (A) TYR. (B) TYRP1. (C) DCT. (D) β-actin.

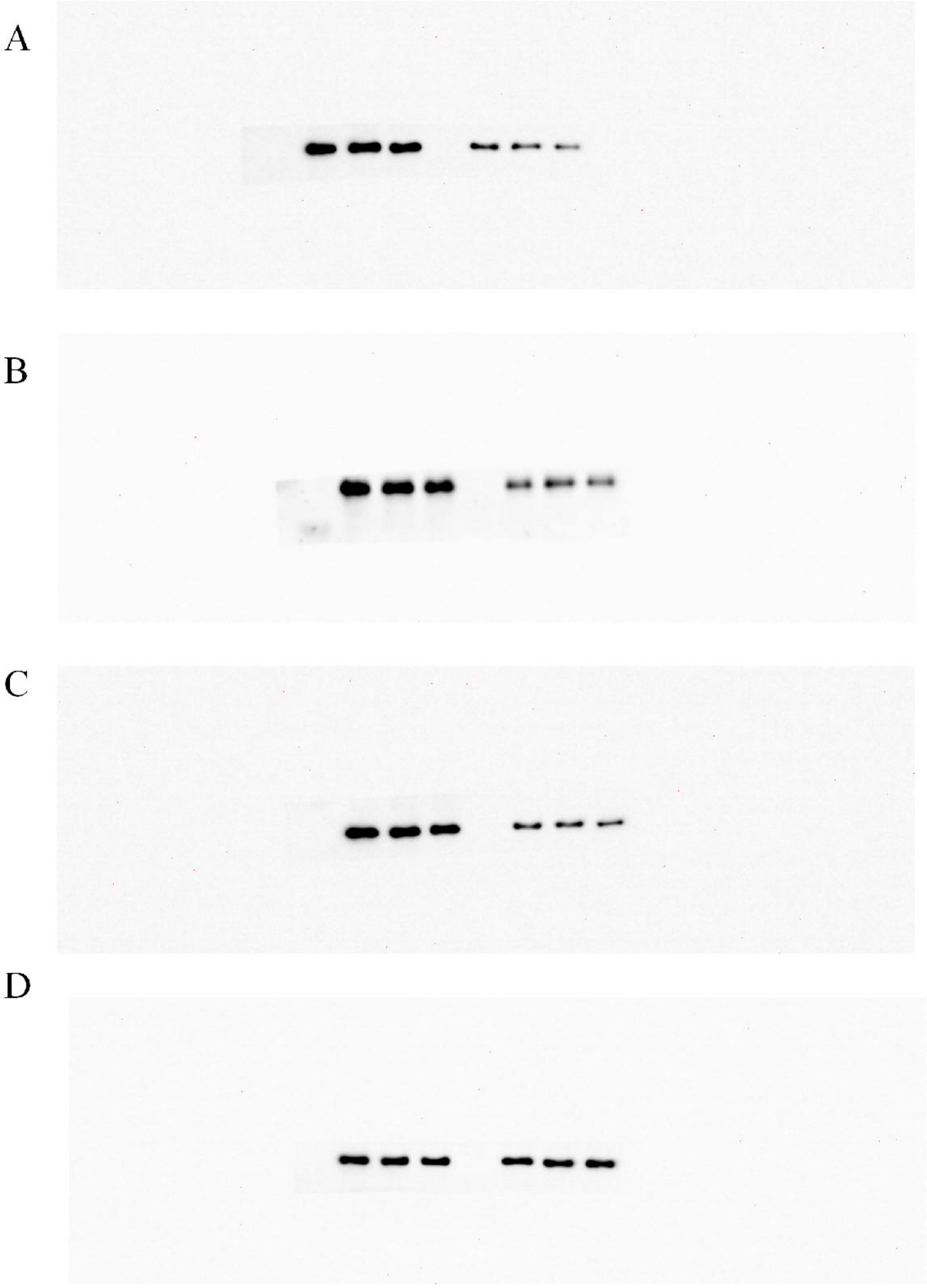

Fig.S6 The original electrophoretic gel results of TYR, TYRP1, and DCT protein expression in melanocytes after transfection with ssc-miR-221-3P. (A) TYR. (B) TYRP1. (C) DCT. (D) β-actin.
